# Mechanical Characterization of High-Performance Steel-Fiber Reinforced Cement Composites with Self-Healing Effect

**DOI:** 10.3390/ma7010508

**Published:** 2014-01-20

**Authors:** Dong Joo Kim, Seok Hee Kang, Tae-Ho Ahn

**Affiliations:** 1 Department of Civil and Environmental Engineering, Sejong University, 98 Gunja-Dong, Gwangjin-Gu, Seoul 143-747, Korea; E-Mails: djkim75@sejong.ac.kr (D.J.K.); shkang1985@gmail.com (S.H.K.); 2 Innovative Construction Materials Engineering, Institute of Industrial Science, The University of Tokyo; 4-6-1 Komaba, Meguro-ku, Tokyo 153-8505, Japan

**Keywords:** self-healing, fiber reinforcement, bond strength, composite, crack detection

## Abstract

The crack self-healing behavior of high-performance steel-fiber reinforced cement composites (HPSFRCs) was investigated. High-strength deformed steel fibers were employed in a high strength mortar with very fine silica sand to decreasing the crack width by generating higher interfacial bond strength. The width of micro-cracks, strongly affected by the type of fiber and sand, clearly produced the effects on the self-healing behavior. The use of fine silica sand in HPSFRCs with high strength deformed steel fibers successfully led to rapid healing owing to very fine cracks with width less than 20 μm. The use of very fine silica sand instead of normal sand produced 17%–19% higher tensile strength and 51%–58% smaller width of micro-cracks.

## 1. Introduction

**Figure 1.**
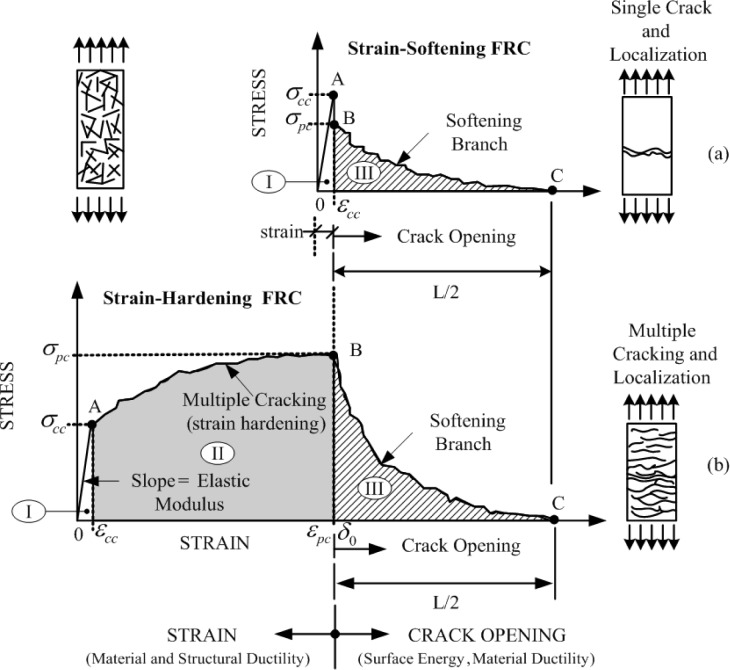
Typical tensile strain softening and hardening behavior of FRC and high-performance steel-fiber reinforced cement composites (HPSFRC) reprinted with permission from [1].

High-performance steel-fiber reinforced cement composites (HPSFRCs) are characterized by their unique strain hardening tensile behavior and multiple micro-cracking behavior, as illustrated in Figure 1 [1]. HPSFRCs are expected to be favorable for robust, tough, and durable civil infrastructure [2–5] based on their high mechanical resistance and energy absorption capacity. In addition, the application of HPSFRCs to civil infrastructure is expected to enhance the durability of infrastructure based on the very fine width of micro-cracks of HPSFRCs. Thus, the use of HPSFRCs in civil infrastructure as structural members or repairing (or retrofitting) materials is strongly expected to improve the resilience of civil infrastructure under severe mechanical and environmental loading conditions.

Recently, the crack self-healing behavior of engineered cementitious composites (ECCs), a type of high-performance fiber reinforced cementitious composites with tensile strain hardening behavior, has been intensively reported. The self-healing behavior of ECCs is mainly based on very fine width of their multiple micro-cracks [6–8]. The crack self-healing of ECCs was obtained by the precipitation of calcium carbonate [9] within the very fine width of micro-cracks. In fact, Yang *et al.* (2009) experimentally demonstrated the main product of self-healing is calcium carbonate [7] by using energy-dispersive X-ray spectroscopy (EDS) analysis. Homma *et al.* (2009) investigated the self-healing behavior of various fiber reinforced cement composites (FRCCs) with different types of fiber and they reported that FRCCs with different types of fiber produced different self-healing speed and capacity [10]. Polyvinyl alcohol (PVA) and Polypropylene (PP) fibers added in mortar matrices or concrete generally facilitated the formation of calcium carbonate on the surface of fibers as well as on the surface of crack with small width owing to their very fine diameter whereas steel fibers generated healing products only on the surface of very fine cracks.

The self-healing capacity of FRCCs and HPSFRCs is thought to be much favorable for the higher durability of civil infrastructure using them. However, most previous studies have focused on crack filling (or sealing) for the purpose of water proofing and on the healing behavior of ECCs with polymer fibers such as polyvinyl alcohol (PVA) or polyethylene (PE) fibers [6–8,10–13]. The effect of self-healing on the mechanical resistance of HPSFRCs has been rarely reported whereas that of ECCs has been intensively investigated in a few references [7,8,10]. In particular, has still not fully been discovered whether the first and post cracking tensile strength of HPSFRCs can be fully recovered or maintained because steel fibers are highly susceptible to corrosion.

Ahn and Kishi (2010) mentioned that the self-healing behavior of FRCCs or ECCs would be beneficial for water proofing [14] only if there was no recovery in their mechanical resistance. In order to obtain the mechanical recovery after healing of the pre-damaged FRCCs or HPSFRCs, the pullout resistance of bridging fibers should be maintained or not be reduced right after pre-damaging the interface or after healing the interface between fiber and matrix. The post cracking tensile strength of HPSFRCs is mainly dependent upon the pullout resistance of fibers. However, there is little information about the pullout resistance of pre-debonded fibers after healing.

Thus, this study aims to investigate the self-healing behavior of HPSFRCs. Detailed objectives are as follows: (1) investigate any healing effects on the pullout resistance of predebonded steel fibers; (2) characterize the mechanical recovery of HPSFRCs after healing; and (3) estimate the effect of healing conditions on the mechanical recovery of HPSFRCs under tension.

## 2. Experimental Program

An experimental program was designed to investigate the mechanical recovery of HPSFRCs after healing. Single fiber pullout tests were carried out to investigate any healing effects on the pre-debonded steel fibers by healing for 3, 7, and 14 days. To quantify the effects of healing on the pullout resistance, the ratios of pullout loads between before and after healing were calculated. And, the effects of healing on the mechanical recovery of HPSFRCs were investigated by performing direct tensile tests after healing for 3, 7 and 14 days. The effects of fiber and sand type on the mechanical recovery of HPSFRCs were compared by analyzing tensile parameters such as first and post cracking tensile strength, strain capacity and averaged width of micro-cracks. Finally, the healing speed and the morphology, shape, and size of rehydration products within cracks were investigated using digital 3D microscopy and electron probe microanalysis (EPMA).

### 2.1. Materials and Specimen Preparation

**Figure 2.**
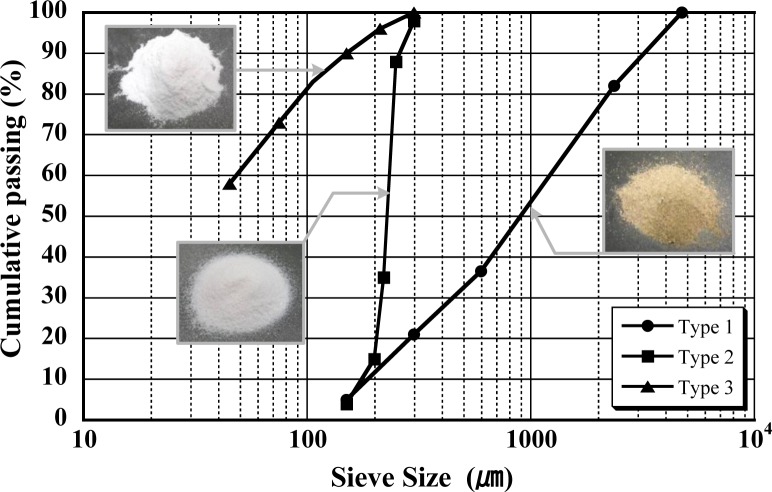
Types of sand.

In single fiber pullout tests, three types of steel fibers—smooth (S-), hooked (H-), and twisted (T-)—were investigated because they have different pullout mechanism. And, two types of sand with different sizes, normal crushed sand (type 1) and very fine silica sand (type 3), were employed in direct tensile tests whereas one type of sand, fine silica sand (type 2), was used in single fiber pullout tests in order to focus on the effect of different pullout mechanisms on the pullout resistance of pre-debonded fibers after healing. The cumulative passing ratios of three different types of sand were illustrated in Figure 2. The composition and compressive strength of the mortar matrix are provided in Table 1 while the properties, including geometry, strength, stiffness and density, of steel fibers investigated are given in Table 2. In the direct tensile test specimens, the amount of those steel fibers added in mortar matrices was 2% by volume. T-fibers had a triangular section with three ribs per 30 mm fiber length. A mortar mixer was used to prepare the cement mixture for both the single-fiber pullout and tensile tests. The detailed procedure for preparing both pullout and tensile specimens can be referred in Kang *et al.* (2012) [15]. Cement, fly-ash, silica fume and sand were dry mixed for 3 to 4 min and then superplasticizer was added with 5 min further mixing. In preparing single-fiber pullout test specimens, the embedment length and inclination angle could be successfully controlled using a device for holding fibers. For preparation of tensile specimens, fibers were distributed by hand when the mortar showed suitable workability and viscosity for uniform fiber distribution. Then, the mortar mixture with fibers was carefully placed in molds using a wide scoop. All specimens were covered with plastic sheets and stored at room temperature for 24 h prior to demolding. After demolding, the specimens were placed in a water tank at room temperature for an additional 2 weeks in a laboratory.

### 2.2. Test Methods and Procedures

**Figure 3.**
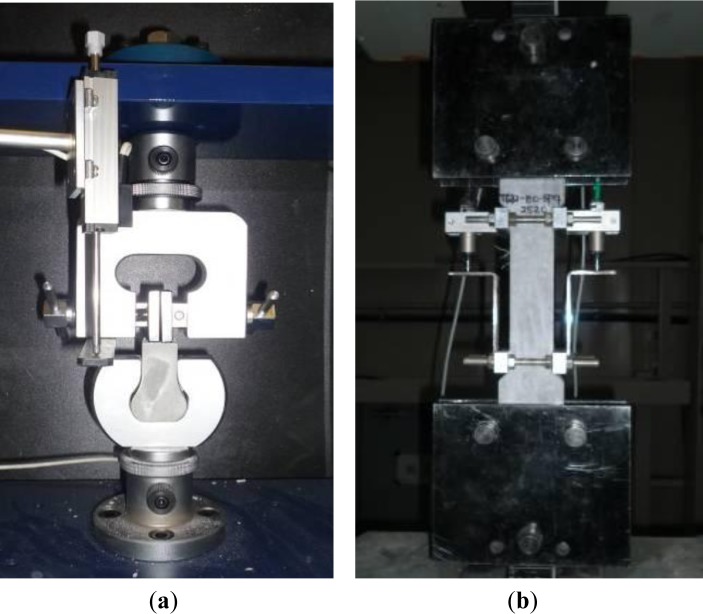
Test set-ups. (**a**) Pullout test; (**b**) Tensile test.

The detailed procedure for single-fiber pullout and direct tensile tests can be found in Kang *et al.* (2012) [15] and the test set-ups for them were provided in Figure 3. Briefly, a fiber was embedded at the center of a pullout test specimen with a section of 25 × 25 mm^2^; and, the embedment length of the fiber was maintained as 15 mm, which was a half of fiber length. To produce interfacial debonding, the fiber embedded was first pulled out until 1 mm slip at 28 days. The 1 mm slip was applied to ensure interfacial debonding along the entire embedded length of fiber by fully activating the mechanical interaction between the fiber and the matrix. Since H-fibers, among the fibers investigated, generally showed the highest pullout resistance when the slip reached 0.5–0.8 mm, 1-mm slip was applied. Then, the pre-damaged pullout specimens were stored in air or water for 3, 7, and 14 days healing. To obtain water healing conditions, pH 7 was maintained to consider continuous water leakage. After the healing periods, the pullout specimens were dried for 1 day at room temperature with 50%–60% relative humidity and then re-pulled out until complete fiber pullout.

In direct tensile tests, to investigate the mechanical recovery of HPSFRCs after healing, all tensile specimens were pre-tensioned at the age of 90 days until the tensile strain reached 0.3% to generate multiple micro-cracks in the specmens. Then, the pre-cracked tensile specimens were stored in both water and air for 3, 7, and 14 days, respectively. As in the pullout test specimens, for water healing, pH 7 was also maintained in the direct tensile test program. After healing in water, the specimens were removed from a water tank and dried for 1 day in air. Then, the healed specimens were re-tensioned using the tensile test set-up shown in Figure 3b. To observe the damaged interface between the fiber and the matrix after fiber pullout and the morphology and shape of the healed materials according to the crack width, the pre-damaged specimens were cut using oil as a lubricant and polished. A digital 3D microscope (Keyence VHX-1000, Tokyo, Japan) and electron probe microanalyzer (Shimadzu EPMA-8705, Tokyo, Japan) were used to investigate the morphology of the cross section containing steel fibers and for the chemical analysis of self-healing products. 3D X-ray microcomputer tomography (micro CT) (Bruker, Delft, The Netherlands) was also used to investigate the cracks and damaged area at the interface after pullout.

## 3. Results and Discussion

### 3.1. Pullout Resistance of Pre-Debonded Steel Fibers after Healing

**Figure 4.**
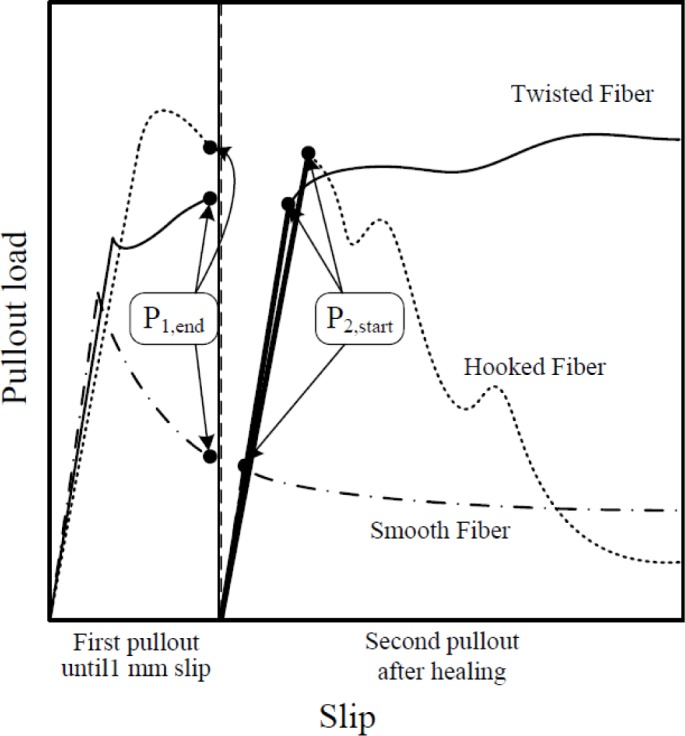
Pullout behavior of high-strength steel fibers after pre-damage up to 1-mm slip.

The mechanical recovery of HPSFRCs is definitely dependent upon the pullout resistance of pre-debonded fibers after healing. Firstly, to quantitatively estimate the effects of healing on the pullout resistance of pre-debonded steel fibers, the maximum pullout load (*P*_2,max_) during the second pullout was compared with the maximum pullout resistance of fibers (*P*_1,max_) during first pullout. To quantify the effects, the ratio, *R*_M_, between *P*_2,max_ and *P*_1,max_ was calculated. Higher *R*_M_ is thought to generate higher post cracking tensile strength of pre-damaged HPSFRCs after healing because the post cracking tensile strength, σ_pc_, strongly depends on the maximum pullout resistance of the fiber. And, to investigate whether the de-bonded interface between fiber and matrix can be healed, the ratio, *R*_H_, between *P*_2,start_ and *P*_1,end_ was calculated. The ratio, *R*_H_, was also believed to effect on the first cracking strength, σ_cc_, of HPSFRCs after healing. Both *P*_2,start_ and *P*_1,end_ are identified in the pullout stress *versus* slip curves as shown in Figure 4: *P*_1,end_ is the end point during the first pullout while *P*_2,start_ is the limit of proportionality during the second pullout.

**Figure 5.**
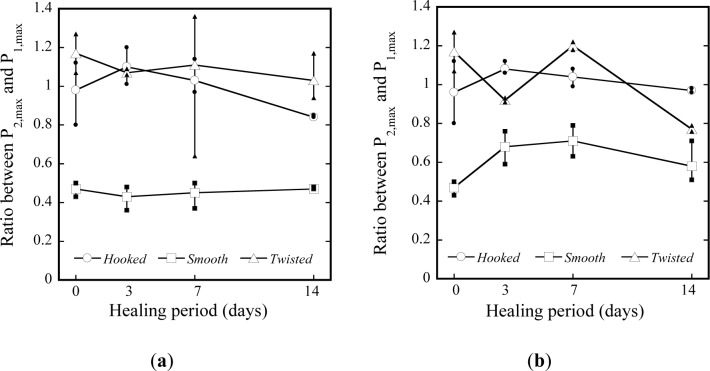
Ratio between *P*_1,max_ and *P*_2,max_ according to types of fiber and healing condition. (**a**) Air; (**b**) Water.

The *R*_M_ of deformed, T- and H-, steel fibers were much higher than that of smooth steel fibers as shown in Figure 5: those of deformed fibers were close to 1.0 and ranged between 0.77 and 1.20 whereas those of S-fibers varied between 0.43 and 0.71. The difference arose from their different pullout mechanisms. The *R*_M_ of deformed steel fibers was not clearly influenced by the condition and age of healing owing to the different pullout mechanisms whereas that of S-fibers was slightly enhanced as the age of water healing increased.

**Figure 6.**
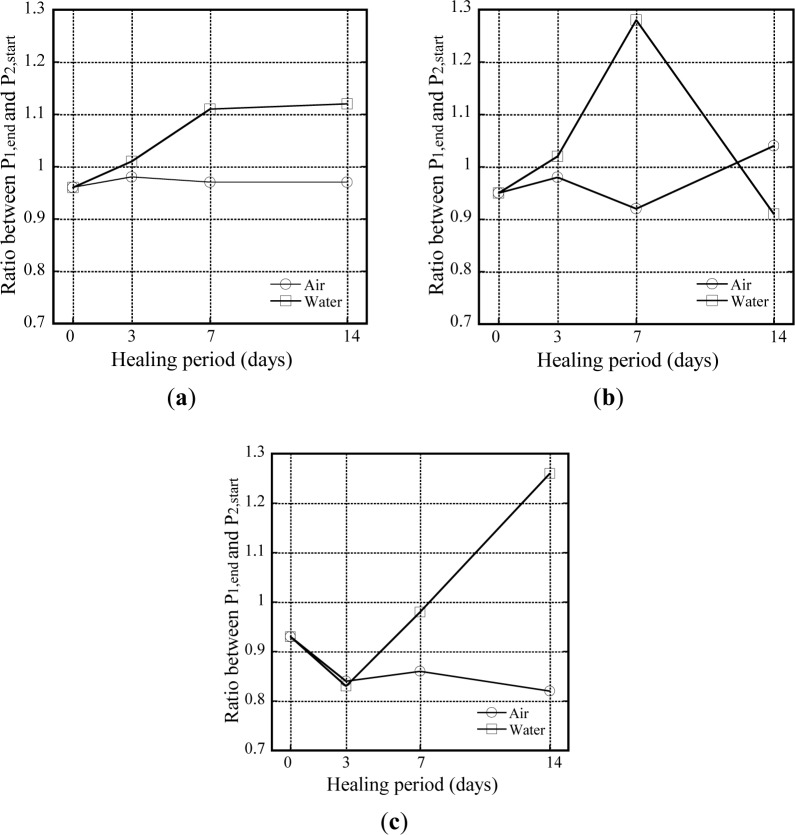
Ratio between *P*_1,end_ and *P*_2,start_ according to types of fiber and healing condition. (**a**) Hooked fiber; (**b**) Smooth fiber; (**c**) Twisted fiber.

It was hard to find significant effects of healing on *R*_H_, as shown in Figure 6 because of the difficulty in water penetration into the interface between the fiber and the matrix. The initial healing or sealing at the interface might prevent further water penetration into the interface. As shown in Figure 6, water healing produced slightly higher values of *R*_H_ than air healing, although the values differed slightly according to the type of fiber. The *R*_H_ of T-fibers subjected to water healing increased from 0.9 to 1.3 as the age of healing increased from 3 to 14 days. The *R*_H_ of S-fibers subjected to water healing also increased until 7 days; however, the S-fiber was corroded after 14 days of water healing. The higher *R*_H_ of water healing might arise from continual hydration rather than interfacial healing of the debonded zone. Further investigation is needed to quantify the effect of interfacial healing alone on the *R*_H_.

**Figure 7.**
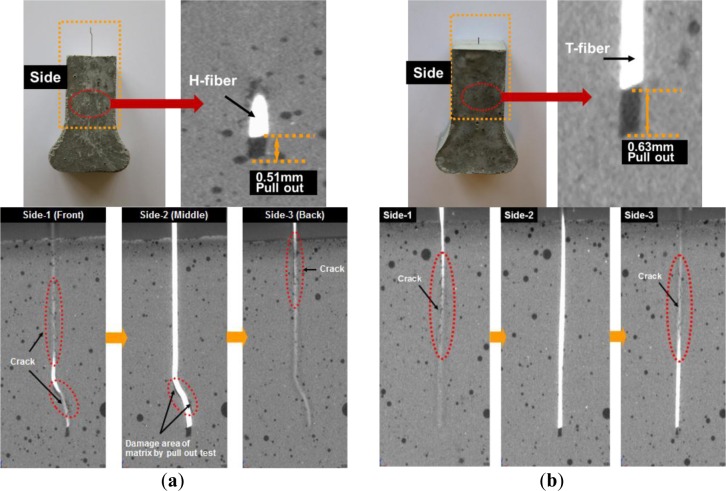
Interfacial cracking behavior of fibers after fiber pullout. (**a**) Hooked fiber; (**b**) Twisted fiber.

The interfacial cracking behavior during the fiber pullout could be successfully captured by 3D X-ray micro CT, as shown in Figure 7 for the deformation of H- and T-fibers in the matrix after 1-mm fiber pullout. The end hook of the H-fiber was significantly straightened during 1 mm slip, as shown in Figure 7a; the damaged area of the matrix owing to the straightening of the end hook could be clearly observed; furthermore, it was observed that the H-fiber slipped by only 0.51 mm from the matrix due to stretching of the fiber although 1-mm slip was applied. In contrast, the T-fiber in the matrix showed interfacial cracking behavior with various tiny cracks with a zigzag path due to the twisted geometry, as shown in Figure 7b, although it did not show as severe damage at the interface as did the H-fiber. This figure suggested that the T-fiber should be untwisted to pull it out; furthermore, the T-fiber slipped by only 0.63 mm although a 1 mm slip was applied.

### 3.2. Effect of Fiber and Sand Types on the Tensile Behavior of HPSFRCs

**Figure 8.**
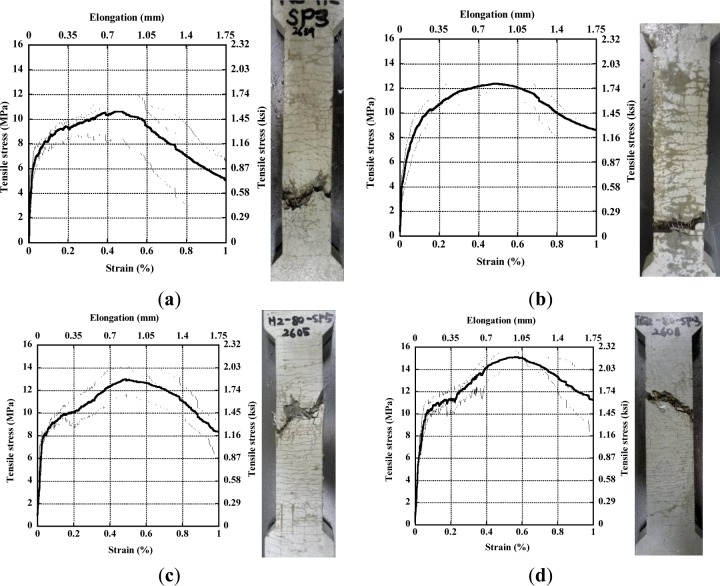
Tensile stress–strain curve of HPSFRCs according to types of fiber and sand. (**a**) H-fiber 2% in matrix A; (**b**) T-fiber 2% in matrix A; (**c**) H-fiber 2% in matrix B; (**d**) T-fiber 2% in matrix B.

Figures 8a–d respectively show the tensile stress-strain curves for each series with H-fiber 2% in matrix A, T-fiber 2% in matrix A, H-fiber 2% in matrix B, and T-fiber 2% in matrix B. All the test series produced tensile strain hardening behavior accompanied with multiple micro-cracks as shown in Figure 8, although the tensile parameters differed according to the types of sand and fiber. Table 3 provides averaged values for the typical tensile parameters such as first cracking strength (σ_cc_), post cracking strength (σ_pc_), strain capacity (ε_pc_), number of cracks within gauge length, average crack spacing, and average crack width. First cracking strength was identified at the limit of proportionality while the post cracking strength and strain capacity were determined at maximum tensile strength point in the stress *versus* strain curve. The number of multiple micro-cracks was counted from both front and back surfaces and then averaged. And, the average crack width was calculated using the average number of cracks within the gauge length and the elongation at the maximum tensile strength.

The average values of tensile parameters provided in Table 3 were averaged from at least three specimens. The series of matrix B containing type 3 silica sand produced more number of micro-cracks and smaller width of those cracks. T-fibers generally generated higher tensile strength, more number of multiple micro-cracks, and smaller width of crack than H-fibers. Thus, the use of T-fibers is expected to produce better conditions for self-healing than H-fibers. Moreover, the very fine silica sand (type 3) generated 17%–19% higher tensile strength and 51%–58% smaller width of crack.

### 3.3. Effect of Fiber and Sand Types on the Self-Healing Behavior of HPSFRCs after Healing

**Figure 9.**
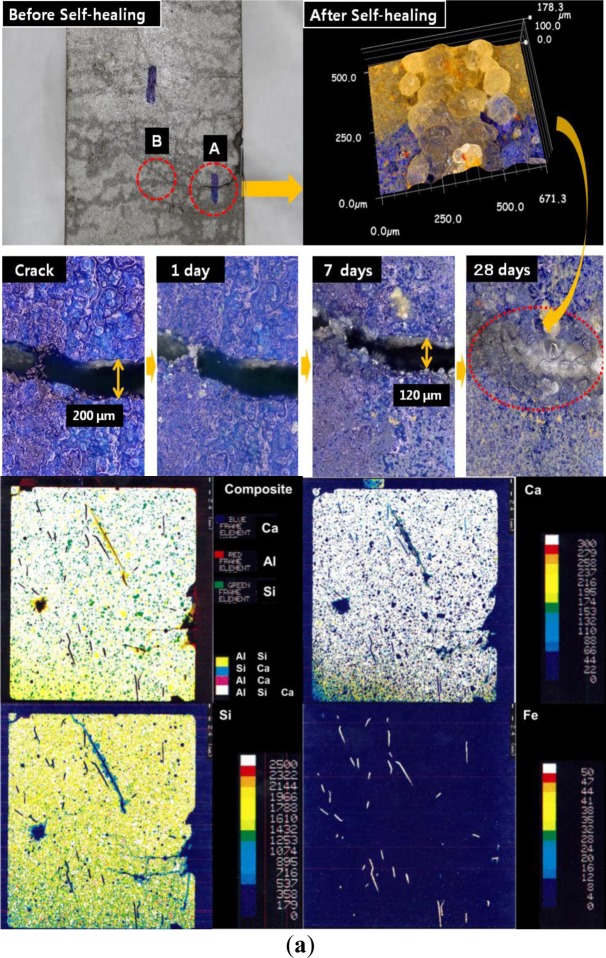

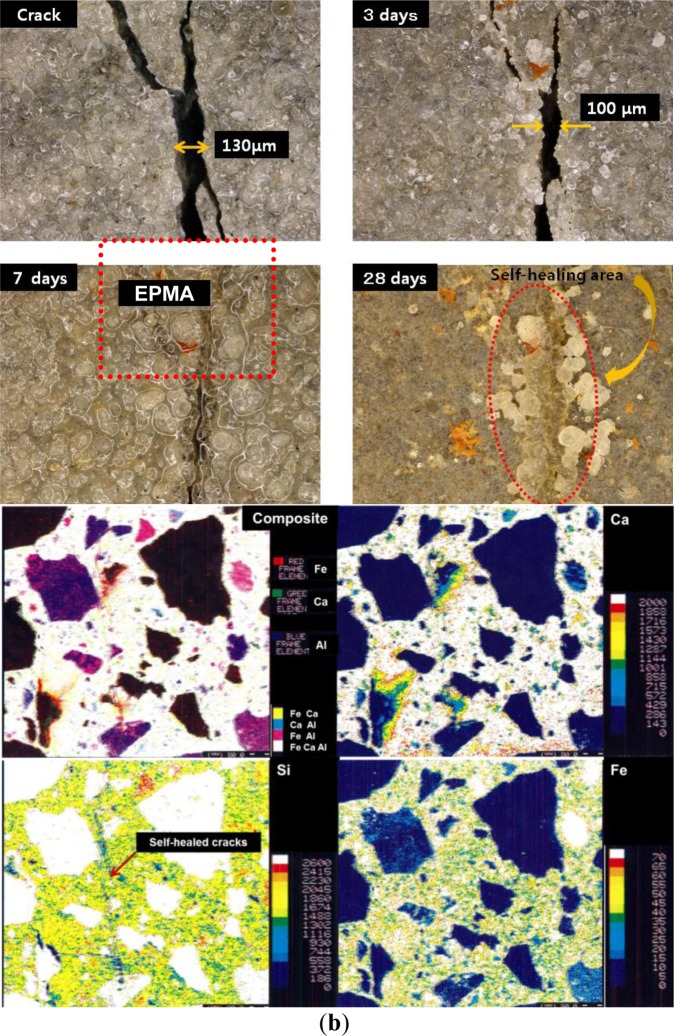
Process of self-healing in matrix A with 2% hooked fiber. (**a**) Zone A with 200-μm crack width (electron probe microanalysis (EPMA) after self-healing); (**b**) Zone B with 130-μm crack width (EPMA after self-healing).

Figure 9a,b show the different healing speeds according to the crack width in pre-cracked HPSFRC with 2% H-fibers in matrix A after water healing. The crack with 200 μm width did not heal completely at the early stage (area A), as shown in Figure 9a; however, that with a width less than 100 μm healed considerably at the early stage through the filling of the crack with re-hydration products, as shown in Figure 9b. Furthermore, the crack was closed by the formation of self-healing products after re-curing for 7 days. The EPMA result in Figure 9a shows the entire self-healed area of the cracked specimen. It comprised different phases between the original and the self-healing zone. In particular, re-hydration products were mainly found to comprise calcium alumina silicate materials, as shown in the X-ray mapping results from the polished section (around 1–2 mm depth from the surface). This self-healing phenomenon seems to be related to crystallization by aluminosilicate with calcium ions based on unreacted fly ash. However, in the case of area B, the chemical compositions were found to be different as shown in the X-ray mapping results relative to previous results for the formation of calcite. X-ray spectra obtained from these phases revealed particular trends in the chemical composition as the formation of modified calcium alumina ferric salts and calcite phases proceeded. In this case, some Fe^2+^ and Fe^3+^ ions in the presence of steel fibers appear to have led to the formation of calcium ferric salts between cracks.

**Figure 10.**
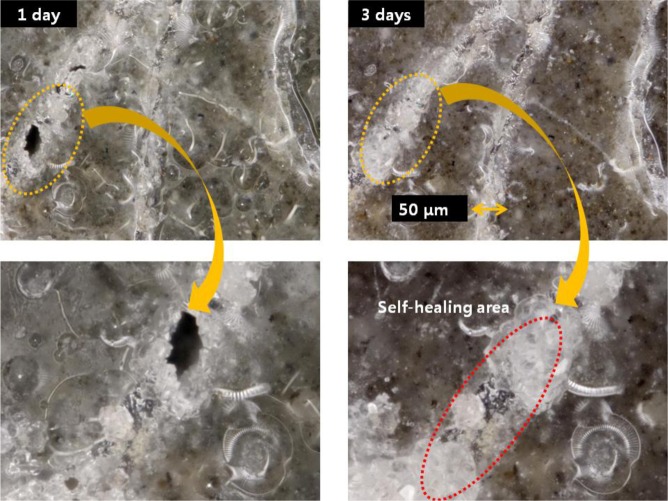
Process of self-healing in matrix A with 2% twisted fiber.

**Figure 11.**
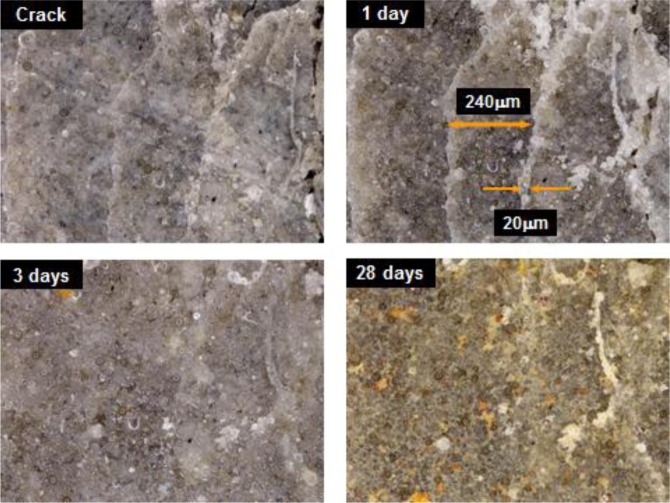
Process of self-healing in matrix B with 2% twisted fiber.

Rust generally forms in the presence of water and oxygen by the following reaction: Fe^2+^ + 2H_2_O ⇌ Fe(OH)_2_ + 2H^+^, Fe^3+^ + 3H_2_O ⇌ Fe(OH)_3_ + 3H^+^. Moreover, some hydration products in the cement matrix with pozzolanic materials have structures similar to that of garnet (3CaO·Al_2_O_3_·3SiO_2_), in which Al^3+^ may be partially replaced by Fe^3+^ and Si^4+^ by 4H^+^ in the solution between cracks during self-healing. These complicated mechanisms are particularly affected by the presence of other ions such as Ca^2+^ and Al^3+^ in the matrix, and they combine with the hydroxides and oxides of iron to precipitate a variety of Ca-Fe-O-OH, Ca-Al-Fe-O-OH, or Ca-Al-Fe-Si-O-OH species. This phenomenon indicates that these products are sensitive to the corrosion, expansion, and bonding properties in the matrix. Therefore, the relationship between self-healing velocity and reasonable amounts of ferric ions should be considered in detail in order to understand the self-healing behavior of HPSFRCs. In other words, the addition of artificial ferric ions with chemical additives might lead to the formation of modified calcium ferric salts, one of the secondary phases, for self-healing. Thus, to improve the physical and chemical stability of these hydration products and precipitated products, the properties of calcium ferric salts in the initial term period should be further investigated; this will be addressed in a future study. As mentioned above, the effect of the crack width on the self-healing behavior of HPSFRCs using T-fibers is clearly shown in Figures 10 and 11. The self-healing capacity of HPSFRCs is confirmed to strongly depend on the crack width. Thus, a suitable sand, fiber, and mineral admixture should be carefully considered in the design of HPSFRCs to maximize their healing capacity.

### 3.4. Influence of Healing Age on the Mechanical Recovery of HPSFRCs

**Figure 12.**
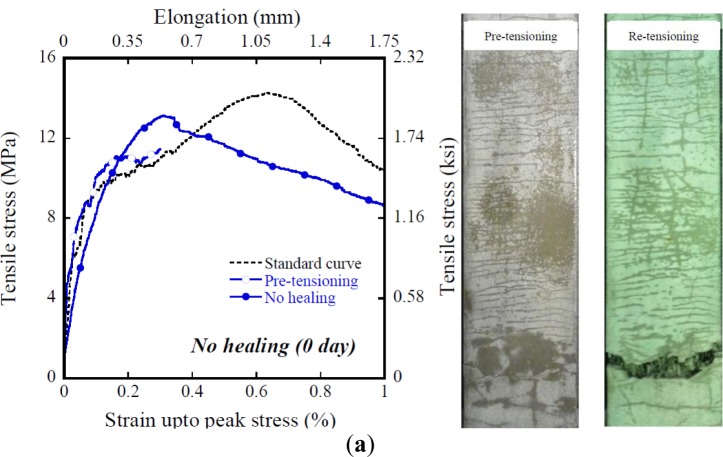

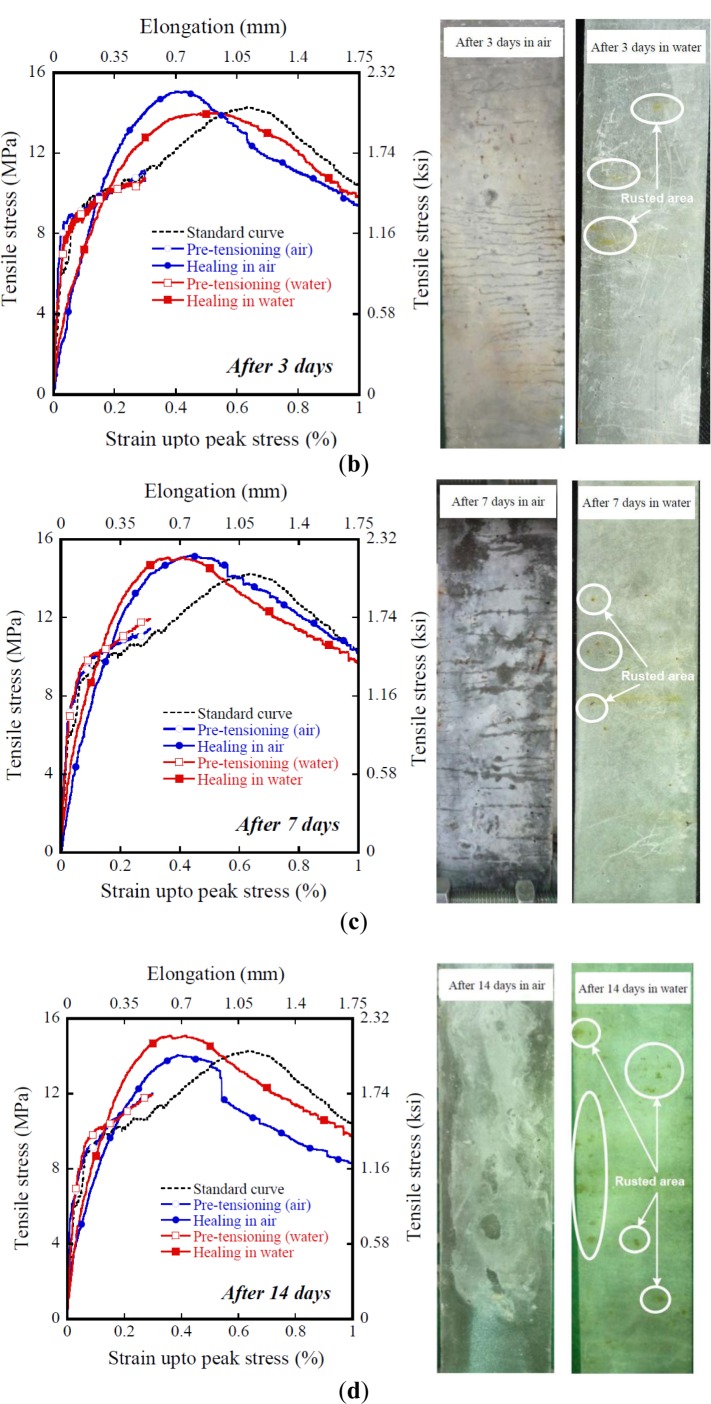
Tensile stress–strain curve of HPFRCCs using T-fiber after healing. (**a**) No healing (0 day); (**b**) after 3 days; (**c**) after 7 days; (**d**) after 14 days.

Table 4 shows the influence of healing age on the mechanical healing capacity of HPFRCCs using T-fibers and very fine silica sand. Figure 12 shows the tensile and cracking behavior of HPFRCCs before and after healing. The standard tensile stress-strain curve shown in Figure 12 was averaged from at least three specimens. The average tensile parameters for the standard tensile stress-strain curve of HPFRCCs with 2% T-fiber and very fine micro silica sand are as follows: σ_cc_, σ_pc_, and ε_pc_ are 9.707 MPa, 15.361 MPa, and 0.519%, respectively.

**Figure 13.**
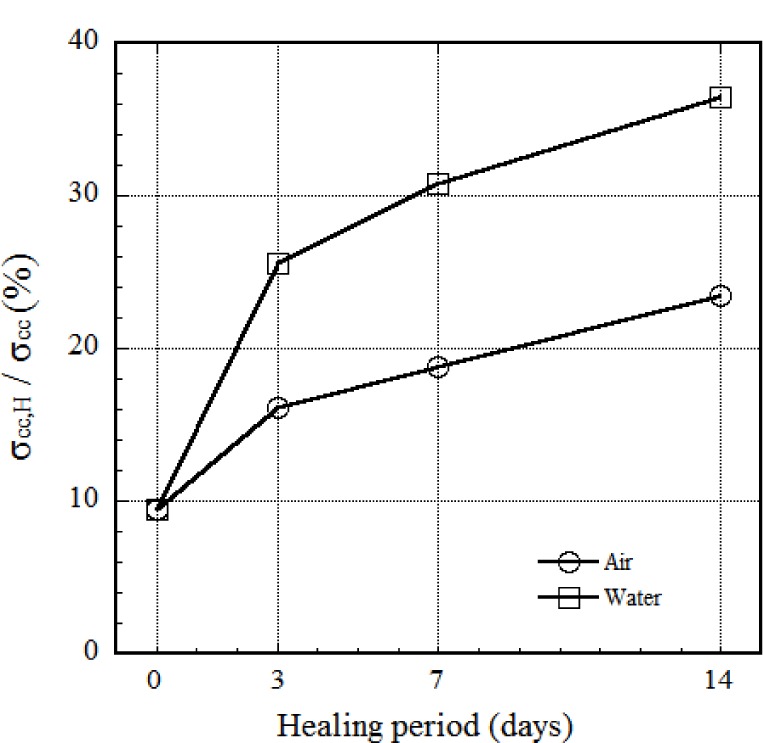
Effect of healing condition on first cracking strength of healed HPFRCCs.

The σ_cc_ of HPFRCCs after healing is much lower than that of the standard curve of HPFRCCs, as shown in Figures 12 and 13. However, the σ_pc_ at the healing period of 7 days is even higher than that of the standard curve of HPFRCC. The ε_pc_ of HPFRCCs after healing is clearly lower than the standard value, as shown in Figure 12 owing to the pre-tensioning until 0.3%.

**Figure 14.**
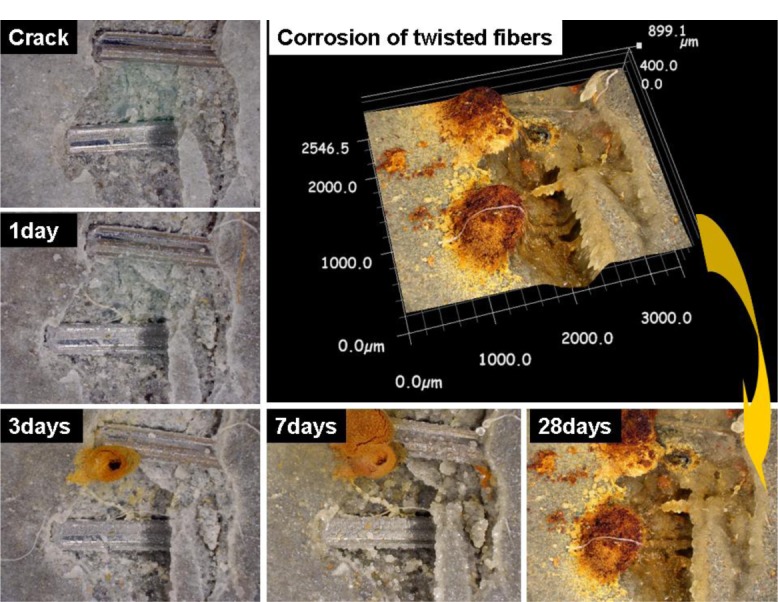
Corrosion process of T-fibers in exposed cracked area with crack width >200 μm.

The influence of healing period on σ_cc_ (first cracking strength before healing) is illustrated in Figure 13 using the ratio between σ_cc,H_ (first cracking strength after healing) and that of the same specimen before healing. As the healing period increases from 0 to 14 days, the ratio increased from 9.4% to 36%, as shown in Figure 13. Although HPSFRCs with T-fibers showed rapid crack healing behavior in the early stage, most healed specimens showed rusted areas on the specimen surface, as shown in Figure 12. Figure 14 shows the corrosion process of the cracked area with T-fibers in the presence of water. In this case, corrosion at the exposed area (over 200 μm) after the pullout test started after 3 days, as shown in Figure 14, and the exposed T-fibers were significantly corroded after re-curing for 14 days. This phenomenon indicates that the healing speed should be further increased to prevent any corrosion of steel fibers in HPSFRCs through micro-cracks.

## 4. Conclusions

The self-healing behavior of HPSFRCs with steel fibers was investigated with a focus on the mechanical recovery of them after healing. The use of finer silica sand with twisted steel fibers generated faster crack self-healing towing to the finer width of micro-cracks lower than 20 μm. The following observations and conclusions can be drawn from the experimental study:

- In the pullout resistance of pre-debonded steel fiber, deformed steel fibers produced higher pullout resistance than smooth steel fibers due to their high mechanical pullout resistance.
- There was no significant healing at the interface between fiber and matrix because the initial healing product at the interface prevented further water penetration into the interface.
- The type of sand and fiber significantly influenced the mechanical (tensile) resistance of HRSFRCs: (1) the use of fine silica sand in HPFRCC instead of normal crushed sand had a favorable effect on the load carrying capacity (17%–19% increase in σ_pc_) and on the crack self-healing behavior (51%–58% decrease in crack width); and, (2) twisted steel fibers produced smaller crack width than hooked steel fibers.
- The self-healing capacity of HPSFRCs is highly dependent on the width of multiple micro-cracks: most multiple micro-cracks, whose widths are smaller than 50 μm, were fully filled by newly formed hydration products after water healing for 3 days. However, some cracks over 200 μm still remained even though the specimen was recurred under water immersion condition for 28 days.
- The self-healing capacity of HPFRCC with steel fibers was significantly affected by ferric ions and various modified calcium ferric salts according to the crack width. In particular, EPMA results revealed particular trends in the chemical composition, such as the formation of modified calcium alumina ferric salts (Ca-Fe-O-OH or Ca-Fe-Al-O-OH species) and calcite phases.
- Regarding mechanical recovery of HPSFRCs after healing, the first cracking strength recovered up to 36% while the post cracking strength recovered almost 100% at 14 days of water healing. However, it was found that the strain capacity after healing was reduced.

The crack sealing of HPSFRCs was observed from microscopic observations; furthermore, the mechanical recovery of HPSFRCs after healing in terms of both the first cracking strength (36% at 14 days of healing) and the post cracking strength (similar to that of the undamaged one) was clearly observed. Future research will aim to increase the crack healing speed and corrosion resistance of steel fibers to prevent their corrosion during the crack healing process.

## Figures and Tables

**Table 1.** Composition of matrix mixtures by weight ratio and compressive strength.

Type	Cement (Type III)	Fly ash	Sand			Silica fume	Super plasticizer	Water	$f_{c}^{'}$ (MPa)
			Type1	Type2	Type3				
Pullout	0.80	0.20	–	1.00	–	0.07	0.02	0.26	103

Tension A	0.80	0.20	1.00	–	–	0.07	0.02	0.26	81
Tension B	0.80	0.20	–	–	1.00	0.07	0.04	0.26	113

**Table 2.** Properties of fibers.

Type	Diameter (mm)	Length (mm)	Density (g/cc)	Tensile strength (MPa)	Elastic modulus (GPa)
Smooth	0.300	30	7.9	2580	200
Hooked	0.375	30	7.9	2311	200
Twisted	0.300*	30	7.9	2428**	200

* Equivalent diameter

** Tensile strength of fiber after twisting.

**Table 3.** Tensile parameters of HPFRCC according to types of fiber and sand.

Matrix	Fiber	σ_cc_*	σ_pc_**	ε_pc_***	Number of cracks	Crack width (μm)	
	
		(MPa)	(MPa)	(%)		Average	Standard deviation
A	H-fiber 2%	7.814	10.738	0.451	18	44.80	14.30
	T-fiber 2%	8.779	12.876	0.450	23	33.76	5.57

B	H-fiber 2%	7.879	12.595	0.546	42	22.91	7.29
	T-fiber 2%	9.707	15.361	0.519	47	19.41	3.92

* First cracking strength

** Post cracking strength

*** Strain capacity.

**Table 4.** Mechanical recovery of HPSFRCs according to different healing conditions.

Healing condition	Healing period (days)	Before healing	After healing			σ_pc,H_
		σ_cc_ (MPa)	σ_cc,H_ (MPa)	σ_pc,H_ (MPa)	ε_pc,H_ (%)	σ_cc_
Air	0	10.098	0.943	13.442	0.409	0.094
	3	8.425	1.361	15.255	0.420	0.161
	7	9.440	1.775	15.904	0.430	0.188
	14	8.904	2.090	14.388	0.436	0.235
Water	0	10.098	0.943	13.442	0.409	0.094
	3	8.888	2.276	14.388	0.491	0.256
	7	9.274	2.859	15.328	0.372	0.308
	14	9.739	3.579	12.846	0.399	0.365
